# Lower urinary tract dysfunction in chronic Chagas disease: clinical and urodynamic presentation

**DOI:** 10.1007/s00345-018-2512-3

**Published:** 2018-10-09

**Authors:** Elsa Bey, Maria Brigitte Paucara Condori, Olivier Gaget, Philippe Solano, Susana Revollo, Christian Saussine, Simone Frédérique Brenière

**Affiliations:** 1 Service d’urologie et de la transplantation rénale, Centre Hospitalier Universitaire de Grenoble, La Tronche, France; 2 Intertryp, IRD-Cirad, Université de Montpellier, Montpellier, France; 3 Instituto de Servicios de Laboratorios de Diagnóstico e Investigación en Salud (SELADIS), Facultad de Ciencias Farmacéuticas y Bioquímicas, Universidad Mayor de San Andrés, La Paz, Bolivia; 4 Centre Hospitalier Annecy-Genevois, Annecy, France; 5 Urological Department, Strasbourg University Hospital, Strasbourg University, Strasbourg, France; 6 Center for Research on Health in Latin America (CISeAL), School of Biological Sciences, Pontifical Catholic University of Ecuador, Quito, Ecuador

**Keywords:** Chronic Chagas disease, *Trypanosoma cruzi*, Bladder, Neurogenic bladder, Lower urinary tract, Urodynamic

## Abstract

**Purpose:**

To describe and give an estimation of the prevalence of urinary disorders in chronic Chagas disease, since most clinical research has been centered on the description of the cardiac and digestive forms.

**Methods:**

To explore this topic, a cross-sectional study was conducted in 137 Bolivian adults of both sexes suffering from symptomatic chronic Chagas disease. All patients presenting confirmed chagasic cardiomyopathy, megacolon or both underwent a urologic symptom questionnaire, uroflowmetry, urinary tract ultrasonography and a creatinine assay. When urinary abnormality was detected, a complete urodynamic study was proposed including cystometry, pressure-flow studies and urethral pressure profile.

**Results:**

Out of all study patients, 35 (26%) had a Chagas cardiomyopathy, 81 (59%) a megacolon, and 21 (15%) a megacolon associated with cardiomyopathy. In all, 63% presented urinary disorders defined by IPSS > 7 and/or ICIQ SF > 1. Among them, 62% were incontinent, mainly by bladder overactivity, and 45% presented grade 2 or 3 renal insufficiency. Of 49 patients, the urodynamic study identified 34 patients with detrusor overactivity (69%), mostly in those with Chagas megacolon. Median bladder functional capacity, urethral closure pressure and bladder compliance had normal values. Moreover, 36% of these patients presented moderate hypocontractility, without significant post-void residual.

**Conclusions:**

This study evidenced lower urinary tract dysfunction in a majority of chronic chagasic patients; those presenting megacolon were more likely to suffer from urinary incontinence. These results strongly suggest including routine urological clinical investigation in chronic Chagas patients, as urinary incontinence due to overactive bladder is frequently observed in this population.

## Introduction

Chagas disease, although it affects about 8 million patients worldwide and is ranked first among parasitic diseases in the Americas by the World Health Organization, is one of the most neglected vector-borne infectious diseases [1]. This disease is caused by the protozoan parasite *Trypanosoma cruzi* and is characterized by an acute phase followed by a chronic one starting about 10 years after the acute phase, and mostly characterized by cardiac pathology (cardiomyopathy) and/or digestive pathology (megaesophagus and megacolon). The first decades following the discovery of the pathology drove an international research effort, mainly concentrated on understanding the typical cardiac and digestive forms. Over the last 20 years, research and interventions have been focused on vector control, which has been a huge success, probably at the price of neglecting these patients who already presented fixed and severe organic lesions due the chronicity of the illness.

This study focuses on a rarely studied aspect of the Chagas pathology: the involvement of the lower urinary tract in chronic Chagas disease. Various studies have previously reported the involvement of the lower urinary tract in experimental chronic Chagas disease [2–6] or in the earliest human studies as described by Koeberle in 1963 on cadaveric specimens [7]. Human symptomatology deriving from bladder, ureteral, and urethral chronic chagasic lesions is as yet widely unknown, and only a few Brazilian studies have intended to describe this clinical and urodynamic presentation [8, 9]. The existing data deriving from animal studies or short human descriptive studies suggest that patients suffering from a digestive form of the disease are more likely to have urinary involvement. In this context, the aim of this cross-sectional study was to assess the prevalence of urinary disorders in all chronic chagasic patients and to describe their symptoms through a complete clinical, biological, ultrasonographic, and urodynamic description. The secondary objective was to highlight the higher putative prevalence of urinary troubles in patients suffering from chagasic megacolon.

## Materials and methods

Between July and October 2017, a cross-sectional study was conducted on patients presenting classical symptoms of chronic Chagas disease. This study was approved by the National Bioethics Committee of La Paz, no. 2/2017, and registered in Clinical Trials with identification number NCT03189056.

### Inclusion and exclusion criteria

**Fig. 1:**
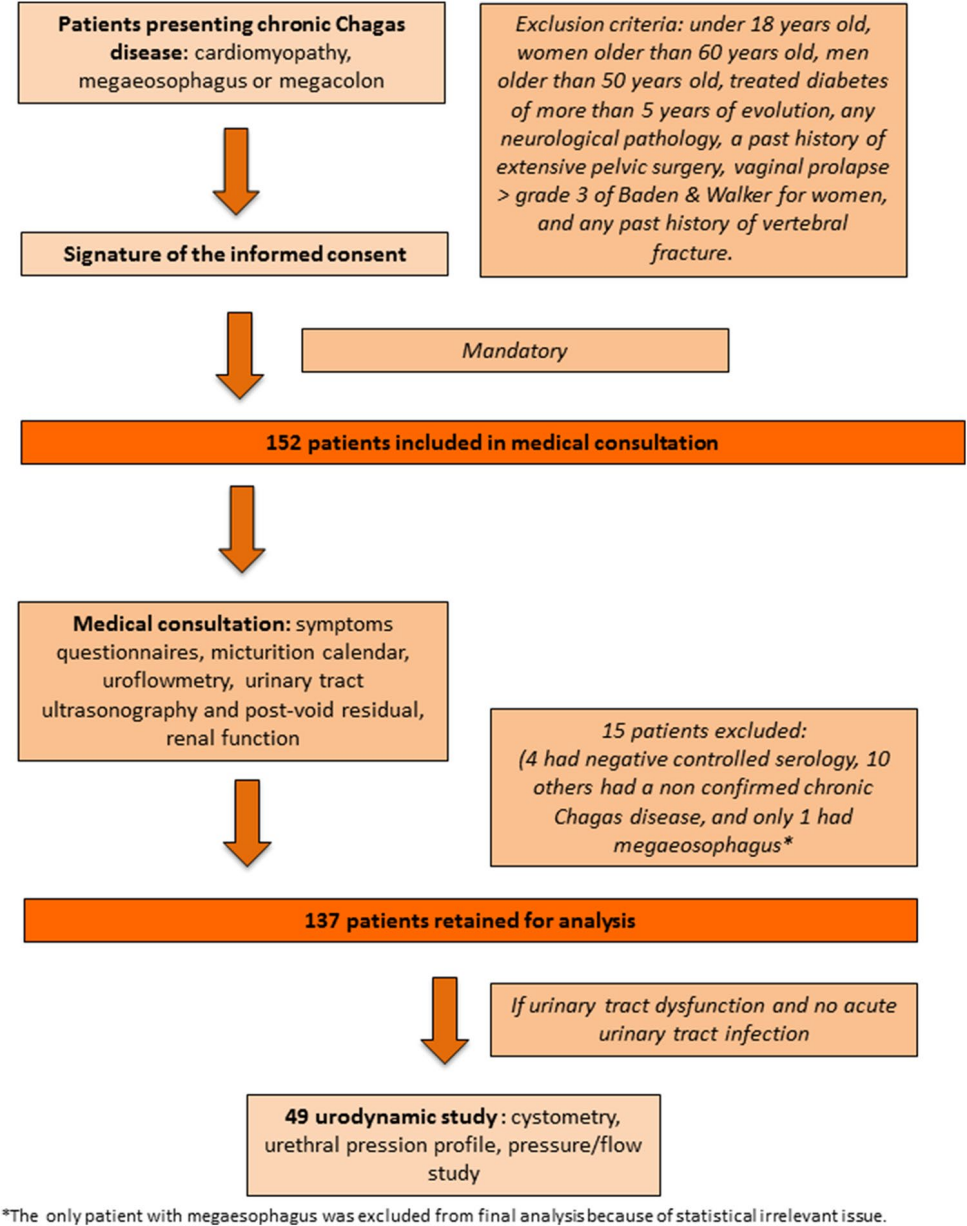
Flow chart of admission and care provided to patients for urological exploration

We included all adult patients presenting confirmed symptomatic chronic Chagas disease: previous positive Chagas serology and chagasic cardiomyopathy, megacolon, megaesophagus or a mixed clinical pattern. The patients included (Fig. 1) had been either previously treated or not for Chagas disease. Patients with chagasic cardiomyopathy presented abnormal electrocardiogram with typical rhythm abnormalities, and/or aneurysm of left ventricle or a pacemaker implant. Patients with chagasic megacolon presented severe constipation, and most of them had a water-soluble contrast enema demonstrating megacolon, a past history of fecaloma or had undergone specific surgery. Chagasic megaesophagus in patients had been confirmed using the barium swallow test. We excluded all women of more than 60 years old and men of more than 50 years old to avoid specific bias due to aging, such as benign prostatic hyperplasia or bladder overactivity and hypocontractility, treated diabetes evolving over more than 5 years, any neurological pathology, a past history of extensive pelvic surgery, vaginal prolapse higher than grade 3 on Baden and Walker for women, and any past history of vertebral fracture. Figure 1 summarizes exclusion criteria applied after clinical examination and questionnaires.

### Recruitment

Patients were addressed by general practitioners/family doctors or by specialists or healthcare centers in villages, after having been informed of the study. Other patients came voluntarily after radio or television information. The recruitment took place in Bolivian cities: La Paz (Hospital de Clinicas), Sucre (Hospital Santa Barbara), Tarija (Hospital San Juan de Dios) and Cochabamba (Hospital Obrero no. 2, Caja Nacional de Salud). Medical attention and exams were totally free of charge. If necessary, translation of the Quechua language was ensured by a local bilingual nurse.

### Protocol

For each patient, we collected past medical history, current treatment, Chagas disease symptomatology and treatment, urological symptomatology using two questionnaires (International Prostate Score Symptom, IPSS and the International Consultation Incontinence Questionnaire Urinary Incontinence Short Form, ICIQ SF), past history of acute urinary retention, upper and lower urinary tract infections, bladder overactivity (ICIQ SF score + micturition calendar), sexuality using the International Index of Erectile Function (IIEF5), and fertility. A micturition calendar over 2 days was obtained. The uroflowmetry was performed for all patients before any previous catheterization (SmartDyn urodynamic system, Albyn medical, Spain, curve obtained via Phoenix software), followed by urinary tract ultrasonography (ultrasonography made with abdominal transducer on a Dakona 2000 system). The curve obtained was analyzed only if voided volume was greater than 150 ml. A blood sample was taken to measure plasmatic creatinine and for Chagas serology in patients with doubtful previous serology. Creatinine was measured using the enzymatic kinetic technique (Teco Diagnostics, Anaheim, CA, USA). Antibodies to *T. cruzi* were detected with two commercial kits, the rapid test Bioline Chagas Ab rapid, (Alere, Waltham, MA, US), and the Chagas *T. cruzi* IgG—ELISA (NovaTec Immundiagnostica GmbH Technologie & Waldpark, Dietzenbach, Germany). Positive serology was confirmed when the two tests were positive; in case of discordance, the indirect hemagglutination kit HAI Chagas was applied (Polychaco, Buenos Aires, Argentina). Patients with negative controlled serology (the three above-mentioned tests) were excluded from the analysis. A complete urodynamic exploration (cystometry, urethral pressure profile, pressure/flow study) was proposed to patients presenting a symptomatic lower urinary tract dysfunction. The sterility of urine was assessed before the examination by negative rapid strip test (Combur 7 test, Roche SAS, Boulogne-Billancourt, France). The urodynamic procedure was not carried out in case of acute urinary infection. Urodynamic exploration was performed on the SmartDyn Urodynamics System (Albyn Medical, Cordovilla Navarra, Spain) using sterile consumables (Bohler II uretral catheter 10 F, abdominal catheter with air balloon, Péters, Cordovilla Navarra, Spain). The urodynamic study was done by water cystometry with filling speed 30 ml/min. The urethral profile was only performed in women because the measurements are biased in men. Electronic curves were obtained using Windows OS, MS SQL Server 2000 database from Phoenix software.

### Clinical criteria for urinary tract dysfunction

Lower urinary tract dysfunction in patients was considered positive for International Prostate Symptom Score (IPSS) > 7 and/or International Consultation on Incontinence Questionnaire Short Form (ICIQ SF) > 1 (Spanish official validated translations were used).

### Statistical analysis

For univariate analyses, we performed the Fisher’s exact test for categorical variables and linear regressions for numerical variables. Significant variables were then included in a multivariate analysis. We studied the association between the presence of lower urinary tract symptoms and the presence of a chagasic megacolon by a multivariate logistic regression analysis, adjusted on various identified confounding factors such as age, sex, and previous specific treatment (categorical variables). A significance threshold of 0.05 was adopted for all statistical analyses. Statistical analysis was performed using the computing environment R [10].

## Results

### Population studied

Out of 152 patients included, a final number of 137 were retained for the analysis (Fig. 1). Table 1 presents the demographic and clinical features for the overall population and for two pathology groups: patients presenting a chagasic megacolon and patients without (cardiomyopathy). Of all patients, only one-third had previously received a complete specific treatment for Chagas disease, mainly with benznidazole, and a high proportion of the others had to stop their treatment prematurely due to allergic reactions. Repeated urinary infections were significantly more frequent in patients presenting megacolon than cardiomyopathy (*p* = 0.02).

**Table 1:** Demographic and clinical features of the study population

Parameters	Overall patients	Cardiomyopathy	Megacolon	*p* value*
Number of patients	137	35 (25.5%)	102 (74.5%)	NA
Median age (IQR)	46 (39–53)	44 (29–60)	46 (39–53)	0.88
Sex ratio F/M	3	1	6	**<** **0.001**
Median weight (IQR)	65 (58–74)	65 (46–99)	65 (58–73)	0.91
Patient who received complete Chagas-specific treatment	44 (32%)	8 (23%)	36 (35%)	0.21
Patients with gastroparesis	60 (44%)	11 (31%)	49 (48%)	0.11
Patients with orthostatic hypotension	87 (64%)	16 (48%)	71 (70%)	0.2
Patients with repeated urinary infections (> 2 cystitis/year or/and > 1 pielonefritis)	31 (23%)	3 (9%)	28 (27%)	0.02
Patients with lithiasis	17 (12.4%)	4 (11%)	13 (13%)	1
Patients with past acute urinary retention	4 (3%)	1 (3%)	3 (3%)	1

*IQR* interquartile range, *NA* not applied

*p values were estimated among the two groups of pathology: cardiomyopathy and megacolon

### Clinical, biological, ultrasonographic, and uroflowmetric results

Table 2 summarizes the results of the patients’ current urologic analysis. The overall prevalence of lower urinary tract dysfunction, defined as IPSS > 7 and/or ICIQ SF > 1, was 62.8% with 95% CI [54.2–71.3%]. The univariate analysis showed a strong association between the existence of a digestive form (megacolon and mixed groups together) and lower urinary tract symptoms (*p* = 0.002). Urinary incontinence was observed in 62% of patients, more frequent in digestive forms than in cases with cardiomyopathy. Erectile dysfunction was observed in one-third of the men, and acquired significant difficulties reaching orgasm was observed in a majority of women. According to the CKD EPI values, renal function was impaired in 45% of the cases. The median value of the maximum flow rate was close to normal. Fifteen patients only presented a maximum flow rate < 15 ml/s (11%), mainly with insignificant post-void residual. The uroflowmetric curve was pathological in 24% of the cases, showing a plateau or intermittent flow. Of 34 patients who completed the voiding diary exam, 11 had severe overactive bladder syndrome with urinary incontinence and more than seven micturitions daily (32%). The median functional volume for these patients was normal at 500 ml. Most of the other patients were ineligible due to illiteracy (> 50% of the cohort).

**Table 2:** Urinary exploration in 137 chagasic chronic patients

Parameters	Number of patients (*N*)			
	Total (137)	Cardiomyopathy (35)	Megacolon (102)	*p* value*
Urinary symptoms				
ICIQ SF > 1	86 (63%)	14 (40%)	72 (70%)	0.002
IPSS > 7	81 (59%)	13 (37%)	68 (67%)	0.003
Clinical voiding dysfunction (Qmax ≤ 15 ml/s + post-void iresidual > 150	4 (3%)	2 (6%)	2 (2%)	0.24
Urinary incontinence	85 (62%)	13 (37%)	72 (70%)	< 0.001
IIEF5 ≤ 16	7 (28%)	3 (21%)	4 (26%)	0.67
Feminine sexual dysfunction	45 (60%)	5 (27%)	40 (63%)	0.21
Hypofertility	15 (12%)	2 (6%)	13 (16%)	0.52
Renal function				
Median of CKDEPI formula, ml/mn (IQR)	91 (82–104)	91 (70–118)	94 (82–103)	0.87
Renal insufficiency ≥ grade 2	62 (45%)	16 (47%)	46 (45%)	0.85
Urine biology				
Leucocyturie	76 (62%)	11 (39%)	65 (69%)	0.007
Proteinurie	6 (5%)	4 (14%)	2 (2%)	0.025
Uroflowmetry				
Median flow max, ml/s (IQR)	22 (18–32)	20 (8–45)	24 (19–34)	0.01
Median post-void residual urine volume, ml (IQR)	20 (5–53)	28 (0–230)	15 (0–54)	0.21
Pathological curve aspect	21 (24%)	8 (26%)	13 (19%)	0.4
Ultrasonography				
Megaureter (dilatation of renal pelvis ≥ 20 mm or ureter ≥ 7 mm)	7 (5%)	1 (3%)	6 (6%)	0.68
Bladder wall thickness > 5 mm	18 (13%)	5 (14%)	13 (13%)	0.78
Micturitional diary				
Median of micturition/day [range]	6 (5–8)	6 (3–12)	6 (6–8)	0.35
Mega-bladder (bladder capacity ≥ 600 cc and ≤ 4 micturition/day)	2	2	0	NA
Functional volume, ml	500 (400–600)	500 (300–900]	500 (400–600)	0.75

*IQR* interquartile range, *NA* not applied

*p values were estimated among the two groups of pathology: cardiomyopathy and megacolon

The multivariate analysis explored the association between lower urinary tract dysfunction and age, sex, previous treatment, and pathology; a strong association was found between the existence of a digestive form and lower urinary tract symptoms (*p* = 0.03, OR 0.36 [0.14–0.92]). The older the patients were, the more they suffered from urinary tract dysfunction (*p* = 0.01, OR 1.06 [1.01–1.12]). Sex and previous specific treatment were parameters not identified as potential impacting factors.

### Urodynamic results

**Fig. 2:**
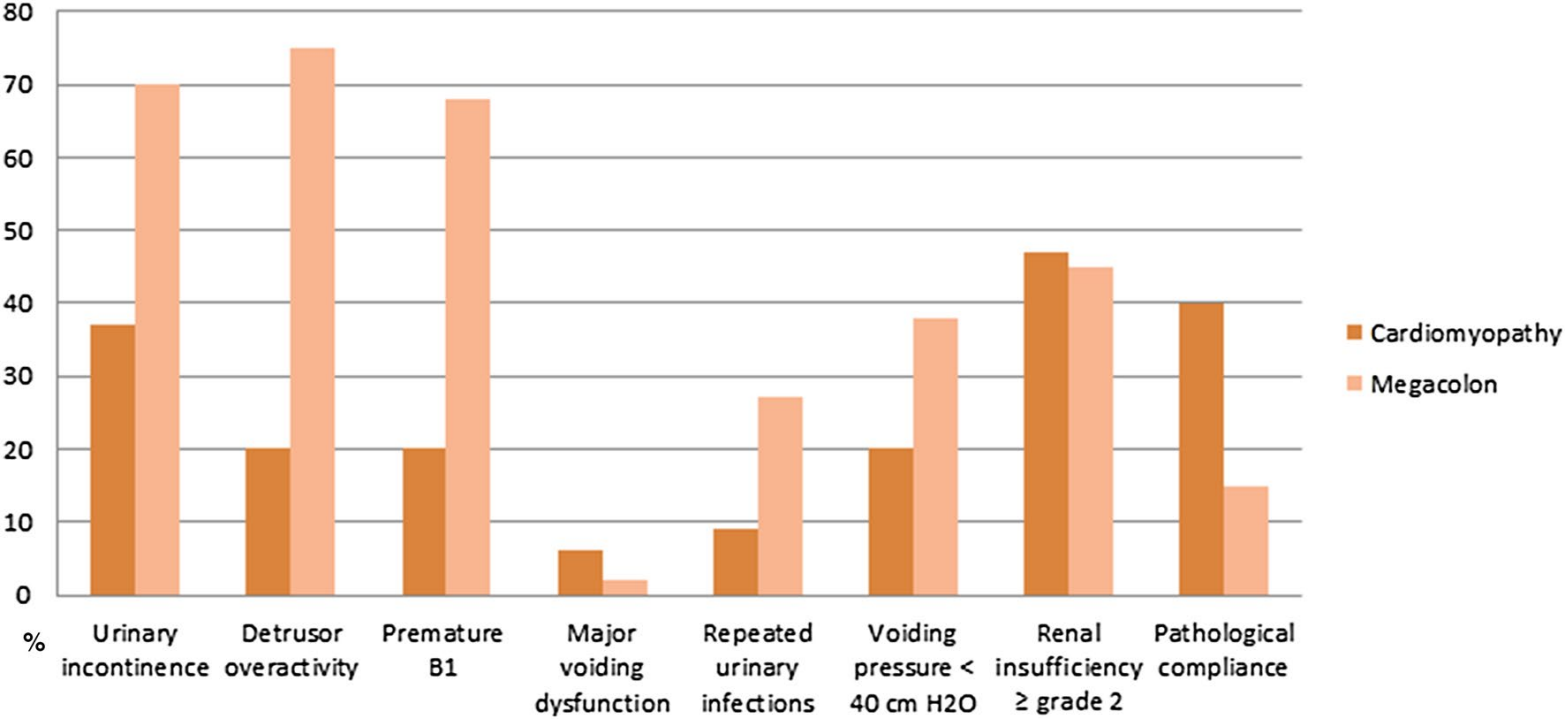
Main results of urological exploration in chagasic patients presenting cardiomyopathy or megacolon. Note: The bar chart represents the percentages of each patient category

Forty-nine urodynamic explorations were achieved in both men and women (Table 3). Many other patients with megacolon or mixed pathology could not be explored due to acute urinary infection at the time of the consultation; they all had chronic severe urological symptomatology and probably would have had pathological urodynamic exploration. The maximum urethral closure pressure on the urethral profile (only women to avoid biased measurements in men) was mainly normal in all groups. The bladder sensation was mainly altered, with premature B1 sensation (first filling sensation < 150 ml with filling speed 30 ml/min) in 68% of the patients presenting megacolon. Median bladder capacity on cystometry was normal-to-low. Detrusor overactivity was found in the majority of the patients (34/49, 69%), very frequently in patients with megacolon (75%), but scarce in patients with cardiomyopathy (20%). The difference between subgroups was statistically significant (*p* = 0.02), confirming the importance of detrusor overactivity in patients suffering from chagasic megacolon. Bladder compliance was altered in eight patients, but no difference between the pathology groups was observed. Low maximum -voiding detrusor pressure < 40 cmH_2_O was less common in patients with cardiomyopathy (20%) than those with megacolon (38%). Figure 2 summarizes the main results.

**Table 3:** Urodynamic results

Parameters	Number of patients (*N*)			
	Total (49)	Cardiomyopathy (5)	Megacolon (44)	*p* value*
Median of urethral closure pressure, cmH_2_O (IQR)	71 (60–83)	32 (22–116)	70 (60–83)	0.99
Median of B1 sensation, ml (IQR)	130 (73–200)	188 (150–222)	119 (68–200)	0.34
B1 sensation < 150 ml	31 (63%)	1 (20%)	30 (68%)	0.05
Median of final filling volume, ml [IQR]	453 (274–500)	475 (388–722)	453 (250–500)	0.15
Detrusor overactivity	34 (65%)	1 (20%)	33 (75%)	0.02
Bladder compliance < 30 ml/cm of water	3 (16%)	2 (40%)	6 (15%)	0.20
Median voiding detrusor pressure, cmH_2_O	54 (31–69)	58 (28–89)	56 (32–68)	0.53
Voiding detrusor pressure ≤ 40 cm of water	16 (36%)	1 (20%)	15 (38%)	1
Presence of abdominal contraction during voiding	12 (24%)	3 (60%)	9 (21%)	0.05

*IQR* interquartile range, *NA* not applied

*p values were estimated among the two groups of pathology: cardiomyopathy and megacolon

## Discussion

To our knowledge, this is the first study providing complete information on lower urinary tract dysfunction in chronic Chagas disease. It shows the very high prevalence (~ 70%) of urinary incontinence in patients suffering from chagasic megacolon, due to overactive bladder in this group of patients. The impact on quality of life is major in this population, the ICIQ SF’s visual scale being ranked > 8/10 by 70% of the patients concerned, confirming the major social impact of this symptomatology.

One limitation of this study could be the lack of a control group: we considered that many very wide studies have, in the past, assessed the prevalence of overactive bladder in the general population, and that a control group representing the general population would in this case be of little interest regarding the existing data already allowing comparison between the general population and our cohort. We may therefore compare our results, finding 63% overactive bladder syndrome and 62% urge incontinence in patients suffering from symptomatic chronic Chagas disease with a median age of 46 years, with the multicenter study of Milsom et al. that found, in more than 16,000 adults older than 40 years of age in six different countries, overactive bladder syndrome in 16.6% and urge incontinence in 5.8% of their population [11]. The very strict care taken in excluding all patients presenting any other pathology possibly responsible for lower urinary tract symptoms, added to the huge difference found in terms of prevalence of overactive bladder syndrome between the two populations, should permit us if not to assess, probably to suspect, despite the lack of control group, a direct causation between chronic Chagas disease and overactive bladder syndrome.

The fact that urinary dysfunction seems to appear mostly in patients suffering from the digestive form of the illness was assumed a few decades ago and has been discussed by several authors [8, 9]. The concomitant alteration of these two neighboring organs can be explained by a common physiopathology. The main histopathological findings in patients presenting chagasic megacolon are degenerative and destructive lesion of the intramural myenteric plexus of Auerbach and focal myositis. Immunological disturbances, by means of a delayed-type hypersensitivity mechanism, are probably the main mechanisms underlying the development of muscular and neuronal alterations, including neuronal destruction with peripheral denervation and focal demyelination [2, 12]. A direct action of the parasite remains possible because infected epithelial cells are frequently found in urine of acutely and chronically infected patients and parasites can be encountered in muscle [3, 13, 14]. The physiopathology of the bladder alterations is therefore neurologic and muscular, direct by parasite action but mainly indirectly mediated by an excessive inflammatory response [2, 13–15]. The parasite’s tissue tropism partially depends on the parasite’s genotype, explaining the geographic pattern of the disease, but not only, given that no strain differences have been detected between infections, whether or not the digestive tract is involved [1]. Involvement of urologic pathology in asymptomatic patients (without cardiac or digestive pathology) is probably very uncommon, but cannot be excluded, opening the field to future studies.

This study proposes a new definition of the typical lower-urinary tract abnormalities in chronic Chagas disease, starting by rejecting the classic term of “chagasic megabladder” commonly used although never justified in any previous study [3, 5]. The bladders’ functional and the urodynamic volume may be normal-to-low in these patients who are suffering much more from overactive bladder than from the supposed enlarged flaccid bladder described in the animal model [3, 5]. Megabladder has indeed never been proved and was described in humans only by Koberlé in 1968 on a cadaveric model [16], which does not reflect the reality of a living and contractile bladder.

In this study, 44.5% of the patients presented G2 level chronic renal disease (CKD EPI between 60 and 90 ml/min). Knowing the commonly normal bladder compliance, the very rare existence of megaureter associated with a known frequent infiltration of the renal tissue by the parasites, we can hypothesize that this alteration is probably mainly due to a direct action of the parasite on renal tissue or via an inflammatory response [17–19].

In their urodynamic study, Rocha et al. [9] found voiding dysfunction and hypocontractile bladders, without any urinary sphincter insufficiency; this result was concordant with Suaid et al.’s work [8]. Our results, showing a normal urinary sphincter without pudendal denervation in all the groups studied, associated with hypocontractility in 30–60% of the patients presenting a digestive form of the illness, therefore have a good external validation.

The alteration of sexual potency, pleasure, and fertility have been assessed previously, demonstrating that Chagas disease was responsible for an alteration of sperm quality and quantity [20, 21].

The question of the impact of treatment on the urinary symptomatology was not the aim of this work, but 32% of the cohort had been previously treated for Chagas disease, and no difference on urinary tract symptomatology was observed between treated and untreated patients. It is today commonly admitted that all subjects presenting chronic *T. cruzi* infection should be treated [22]. However, the impact on the progression of the chronic disease seems worth it only on chronic cardiomyopathy, with contradictory information on the progression of the gastrointestinal form of Chagas disease [1, 23]. The lack of efficiency of the existing etiological treatments should not prevent us from treating the symptom, which is responsible for the alteration of quality of life. Patients suffering from megacolon do benefit from surgery or medical treatment of constipation: the same conclusions apply to urinary incontinence, which has to be sought in these patients and symptomatically treated if found.

Anticholinergics and mirabegron are both effective treatments, with a good security profile, commonly used all over the world to treat overactive bladder, and should be used to improve the quality of life of the patients concerned. This treatment has to be prescribed taking great care to also improve the digestive comfort of these patients bothered by chronic constipation, because these drugs worsen this clinical aspect. Treatment by botulinum toxin has not yet been studied in this population but would probably be better tolerated than anticholinergic drugs. This therapeutic option is still to be evaluated.

## Conclusion

This study emphasizes the importance of urinary incontinence by detrusor overactivity in patients with chagasic megacolon, with strong alterations in terms of quality of life. The recommendation to physicians in Chagas endemic areas is to search for urinary incontinence in this group of patients and to propose an adapted treatment while treating constipation.
